# Hierarchical clustering analysis & machine learning models for diagnosing skeletal classes I and II in German patients

**DOI:** 10.1186/s12903-025-06063-6

**Published:** 2025-05-15

**Authors:** Eva Paddenberg-Schubert, Kareem Midlej, Sebastian Krohn, Iqbal M. Lone, Osayd Zohud, Obaida Awadi, Samir Masarwa, Christian Kirschneck, Nezar Watted, Peter Proff, Fuad A. Iraqi

**Affiliations:** 1 Department of Orthodontics, University Hospital of Regensburg, University of Regensburg, Regensburg, 93047 Germany; 2 Department of Clinical Microbiology and Immunology, Faculty of Medicine and Health Sciences, Tel Aviv University, Tel Aviv, 6997801 Israel; 3 Center for Dentistry Research and Aesthetics, Jatt, 4491800 Israel; 4 Department of Orthodontics, University of Bonn, Bonn, D-53111 Germany; 5 Gathering for Prosperity Initiative, Jatt, 4491800 Israel; 6 Department of Orthodontics, Faculty of Dentistry, Arab America University, Jenin, PNA Israel

**Keywords:** Skeletal malocclusion, Cephalometric analysis, Machine learning, Personalized orthodontics

## Abstract

**Background:**

Classification is one of the most common tasks in artificial intelligence (AI) driven fields in dentistry and orthodontics. The AI abilities can significantly improve the orthodontist’s critical mission to diagnose and treat patients precisely, promptly, and efficiently. Therefore, this study aims to develop a machine-learning model to classify German orthodontic patients as skeletal class I or II based on minimal cephalometric parameters. Eventually, clustering analysis was done to understand the differences between clusters within the same or different skeletal classes.

**Methods:**

A total of 556 German orthodontic patients were classified into skeletal class I (*n* = 210) and II (*n* = 346) using the individualized ANB. Hierarchical clustering analysis used the Euclidean distances between data points and Ward’s minimum variance method. Six machine learning models (random forest (RF), K-nearest neighbor (KNN), support vector machine (SVM), linear discriminant analysis (LDA), classification and regression trees (CART), and General Linear Model (GLM)) were evaluated considering their accuracy, reliability, sensitivity, and specificity in diagnosing skeletal class I and II.

**Results:**

The clustering analysis results showed the power of this tool to cluster the results into two–three clusters that interestingly varied significantly in many cephalometric parameters, including NL-ML angle, NL-NSL angle, PFH/AFH ratio, gonial angle, SNB, Go-Me (mm), Wits appraisal, ML-NSL, and part of the dental parameters. The CART model achieved 100% accuracy by considering all cephalometric and demographic variables, while the KNN model performed well with three input parameters (ANB, Wits, SNB) only.

**Conclusions:**

The KNN model with three key variables demonstrated sufficient accuracy for classifying skeletal classes I and II, supporting efficient and still personalized orthodontic diagnostics and treatment planning. Further studies with balanced sample sizes are needed for validation.

**Supplementary Information:**

The online version contains supplementary material available at 10.1186/s12903-025-06063-6.

## Introduction

Orthodontics is pivotal in diagnosing physiological and pathological jaw positions, necessitating precise classification of skeletal class. Due to their high prevalence, the distinction between skeletal class I and II malocclusion is, particularly, interesting to the orthodontist [1]. Effective treatment planning relies on precisely diagnosing the relationship between the maxilla and mandible, essential for devising tailored treatment strategies [1]. Hence, as in all medical disciplines, the orthodontic practitioner needs to perform precise and correct diagnostics and evaluate each patient’s orthodontic treatment needs individually [2]. Dysgnathia, characterized by abnormal jaw relationships, demands individualized approaches, ranging from functional appliances for growing patients to surgical-orthodontic interventions for adults [3, 4].

Orthodontic diagnostics entail comprehensive assessments, including patient history, clinical examinations, dental cast analyses, and radiographic evaluations, such as orthopantomograms and lateral cephalograms [5]. Cephalometric analysis serves as a cornerstone, facilitating the classification of skeletal patterns and delineating the sagittal relationship between the maxilla and mandible [6]. The prevalence of skeletal classes varies across populations, underscoring the importance of accurate diagnosis tailored to specific demographic profiles [6].

### Clinical implications in using the standard of care diagnosis methods vs. artificial-intelligence methods

There are various methods to define a patient’s skeletal class, including ‘classical’ approaches such as the ANB angle described by Riedel and others [7, 8]. A study that was performed by Wellens et al. [9], and examined the ANB angle. The Wits appraisal performance found that the volume under the resulting ROC (Receiver Operating Curve) surfaces (VUS), found that the diagnostic performance of the conventional ANB and Wits was 81.1% for class I, and 80.75% for class II (*P* > 0.05), while when normalizing the measurements, the performance improved significantly to 91%, and 87.2%, respectively (*P* < 0.001).

Besides, there are individualized techniques like the graphical procedure suggested by Fishman [10], the harmony box established by Segner and Hasund [11, 12], or the individualized ANB, which was introduced by Panagiotidis and Witt [13]. Traditionally, skeletal class determination relied on empirical norms, often leading to diagnostic inaccuracies due to neglecting individual craniofacial harmonies [13–15]. Furthermore, the individualized equations were based on specific ethnic populations, like the individualized ANB, which was introduced by Panagiotidis and Witt [13], which was based on 121 patients from the Orthodontics Department in Würzburg, and the recently published study by Paddenberg et al. [16], which was based on 71 Caucasians males and females, and aimed to improve the regression formula of the individualized ANB angle and Wits appraisal. In addition, the individualized equations didn’t fit all the cases included in these equations. For example, the r correlation coefficient that was reported by Panagiotidis and Witt [13] was *r* = 0.808, while the study of Paddenberg et al. [16] reported corrected *R*^2^ = 0.690 for the individualized ANB, and almost perfect corrected *R*^2^ = 0.984 for the individualized Wits appraisal. In summary, the complexity and variability of cephalometric techniques necessitate innovative solutions to improve diagnostic accuracy. In recent years, artificial intelligence (AI), particularly machine learning (ML), has emerged as a promising tool to enhance orthodontic diagnostics [17–20]. ML models offer the potential to analyze cephalometric data efficiently, aiding in landmark detection and treatment planning [17–20]. Nonetheless, the optimal ML model for skeletal class diagnosis remains elusive, with variations in performance across different populations and datasets [18]. While previous research has explored the application of ML models to classify skeletal classes in various populations, comprehensive studies focusing on German cohorts have been scarce [21–23]. This study endeavors to bridge this gap by establishing an ML model specifically tailored to accurately classify German orthodontic patients into skeletal Class I or II [21–23].

Recently, we evaluated skeletal class II and III patients among the Arab population [24] by establishing a machine-learning model for diagnosing skeletal class II and III. We also evaluated skeletal class I and II machine-learning models [25]. Hence, the primary aim of this prospective, multi-centric cross-sectional study was to establish a robust machine learning model to classify German orthodontic patients as class I or II correctly and to overcome the limitations of the traditional methods, especially borderline cases that can be misclassified. Furthermore, in this study, we will validate the machine-learning models applied to Arab patients and thus generalize these models to more than one ethnic group and the general population.

## Material and methods

### Ethical statement

Before collecting the samples, this investigation received ethical approval from the University of Regensburg (approval number 19–1596-101, 13/11/2019). The recruitment of patients considered orthodontic patients of several German specialist offices and the department of orthodontics of the University Hospital Regensburg, Germany, only. The declarations of Helsinki and the ethical guidelines approved by the university’s committee were complied with during the study.

All patients over 18 years old or parents/guardians of those younger than 18 agreed to participate in this quantitative, observational study after receiving detailed information and signing a corresponding informed consent form.

### Data recruitment and cephalometric analysis

This study was based on the pre-treatment lateral cephalograms of German orthodontic patients, which were taken as part of their routine orthodontic diagnostics. During data recruitment, the following inclusion and exclusion criteria were applied. Inclusion criteria were the availability of a pre-treatment lateral cephalogram with a caliper for calibration, demographic information (age, gender), and the presence of either skeletal class I or II, as diagnosed by the individualized ANB of Panagiotidis and Witt. Patients with skeletal class III were excluded from this study. Applying cephalometric analysis, patients were stratified into the skeletal classes I and II. Furthermore, within each group, age and gender-specific subgroups were built according to the following criteria:

Lateral cephalograms were, if necessary, digitized, imported as lossless TIF files into the software ivoris® analyze pro (Computer konkret AG, Falkenstein, Germany, version 8.2.15.110), and calibrated. Then, the method described by Panagiotidis and Witt was used to determine each individual’s skeletal class and to allocate patients into the groups class I and class II:

- Individualized ANB_Panagiotidis & Witt_ [13] = −35.16 + (0.4 × SNA) + (0.2 × ML-NSL).
- Calculated_ANB = ANB_measured_—ANB_individualized_

To avoid distortion of the data by the inclusion of borderline cases, we applied slightly extended limits compared to the definitions of ± 1°, suggested in the original publication:

- Skeletal class I: −1.5° ≤ Calculated_ANB ≤ 1.5°
- Skeletal class II: Calculated_ANB > 1.5°
- Skeletal class III: Calculated _ANB < −1.5°

Then, a complete cephalometric analysis, which was similar to the one of Segner and Hasund [9, 10], was conducted, evaluating skeletal sagittal, skeletal vertical, and dental parameters, which are listed and defined in Supplementary Table 1 and presented in Supplementary Fig. 1A-B.

After calibration, all cephalometric analyses were conducted by two trained raters (SK, EPS). To ensure reproducible cephalometric measurements, interrater- and intrarater-reliability were verified before the main investigation. For this purpose, 50 cephalometric images were randomly chosen and analyzed by two independent raters (SK, EPS). Intrarater-reliability was assessed by the same investigator’s repeated analysis of the lateral cephalograms with a time interval of at least two weeks to avoid bias. Applying the test–retest-technique, interrater and intrarater reliability proved almost perfect, indicated by ranges between 0.92 to 0.99 and 0.90 to 0.99, respectively. Cephalometric measurements were also made to prepare the data set for the primary outcome of this study, i.e., for the establishment of machine learning models for diagnosis of skeletal class I and II.

### Clustering analysis

The clustering algorithm included skeletal class I occlusion and skeletal class II malocclusion patients and then separately for every skeletal class. A scatter plot and dendrogram were produced using the R statistical program to implement the visualization of the cluster analysis results.

In all our clustering calculations, we used the Ward error sum of squares hierarchical clustering described by Ward in 1963 [26]. In this section, we performed hierarchical clustering analysis and decided on the number of clusters according to the dendrogram result. It was acceptable to present the current results with k = 2, and 3 clusters. The same analysis was performed for skeletal class I and II separately.

### Machine learning models

Different machine learning models were applied regarding the number of input variables and the kind of model to identify the best-fitting and most relevant predicting variables. The tested models included random forest (RF), K-nearest neighbor (KNN), support vector machine (SVM), linear discriminant analysis (LDA), classification and regression trees (CART), and General Linear Model (GLM).

RF is a machine learning model that combines the results of several independent decision trees by bagging, i.e., by weighing all single results concerning predefined criteria. Within each decision tree different criteria are applied, chosen randomly, and hence vary between trees [27].

In the KNN method, a new, unknown data point is classified by determining the category of the closest neighboring data points, called K-points, which have been categorized in the predefined data set in advance. K refers to the number of neighboring data points considered in this classification process. During machine learning model testing, the value of “k” was chosen based on the model’s performance, as the “k” resulting in the highest accuracy was selected.

SVM attributes new data points to one of the predefined classes by separating the known data set into groups using a borderline, which is constructed to present the most significant distance to the predefined categories.

In the context of classification, the principle of LDA is the identification of a linear correlation between variables, which are appropriate to discriminate a data set, and the allocation of new data points into one of the predefined groups. For this purpose, linear discriminants are determined, which maximize the distance between separate classes and minimize the variance within each class. In CART, binary decision trees are used to classify new data points by applying predefined numbers and orders of independent variables. Finally, the GLM model is defined by three components: a linear regression equation, a specific error distribution, and a link function, which is the transformation that links the predicted values to the observed values [28]. Generalized linear mixed models extend linear mixed models to address noncontinuous responses, such as binary responses [29].

### Data analysis

Interrater- and intrarater reliability were verified using the test–retest method. All other statistical analyses were performed with the R software platform (https://www.r-project.org/). Finally, 390 patients (70.1%) of the total study collective were used to determine the performance of the different machine learning models regarding their accuracy, kappa, sensitivity, and specificity. Each model’s best-fitting machine learning model (RF, KNN, SVM, LDA, CART) (general model, models 1 to 3) was validated in classifying patients as class I or II by conducting the k-fold cross-validation with k equaling 10.

Statistical significance and high significance were set at *p* < 0.05 and 0.01, respectively.

### Data validation

The best fitting model, which was assessed using mean accuracy in the cross-validation process, was validated using the unseen set and included 30% of the data by comparing the actual skeletal class diagnosis with the machine learning model and calculating sensitivity and specificity. The results were visualized as a confusion matrix.

## Results

### Patients

This study comprised 556 German orthodontic patients stratified into the skeletal class II (*n* = 210) and I (*n* = 346). Both groups presented a mean age of 13 years with a range of 6.6 to 41 years and 5.4 to 53 years in classes I and II, respectively. Further details concerning the demographic information (age, gender) and the patients’ distribution to the different subgroups are shown in Supplementary Table 2A. Supplementary Table 2B shows the cephalometric measurements of patients with skeletal class I and II.

### Borderline cases

The current study categorized patients as skeletal class I when the Calculated_ANB was in the range −1.5—+ 1.5 instead of −1—+ 1. At the same time, skeletal class II patients were determined to be as Calculated_ANB greater than + 1.5 instead of greater than + 1. In the current analysis, 47 patients were in the range of −1.48 up to −1.02 and should be categorized as skeletal class III, according to the original definition; however, they were classified as skeletal class I. In addition, 59 patients were in the range of + 1.02 up to + 1.5. They should have been categorized as skeletal class II according to the original definition, but they were categorized finally as skeletal class I.

### Clustering analysis

Initially, we included all parameters for the hierarchical clustering process and performed the analysis for the whole data. When applying two clusters to our data, Ward’s method results showed that cluster 1 consisted of 299 mixed skeletal class I and II patients. Cluster 2 comprised 257 skeletal class I and II patients (Table 1 & Supplementary Fig. 3). The results of the clustering of both skeletal class I and II, interestingly variated significantly in many cephalometric parameters, as presented in Table 2.

**Table 1:** Shows the hierarchical clustering results summary according to their skeletal classification (I/II). Summary of hierarchical Ward clustering results when using all variables. This table presents the number of patients in each cluster and their classification (clustering for both skeletal classification class I and II), in addition to the number of patients within each cluster when performing the clustering separately for each class, independently

Patients Included	Cluster	Class Calculated ANB		Total
		**I**	**II**	
All	1	199	100	299
	2	147	110	257
Total				556
Class I	1	105	-	105
	2	94	-	94
	3	147	-	147
**Total**		346		
**Class II**	1	-	88	88
	2	-	122	122
**Total**		210

**Table 2:** Shows the results of the hierarchical clustering analysis for skeletal class I and II patients together. Cephalometric parameters and age, descriptive statistics (mean, and standard deviation (SD)) for each cluster. Besides, the table presents the significance levels between the two clusters using t test analysis (NS—not significant, * < 0.05, and ** < 0.01)

Parameter	Class I & II Malocclusion				
	**Cluster 1**		**Cluster 2**		
	Mean	SD	Mean	SD	**Sig t test**
Age	12.90	3.87	13.15	6.31	NS
NL-ML angle [°]	21.44	5.05	25.96	5.87	**
NL-NSL angle [°]	6.35	3.05	9.40	3.39	**
PFH/AFH (%)	69.86	4.10	63.33	4.00	**
Gonial angle [°]	119.39	5.94	123.90	6.19	**
Facial axis	91.24	3.92	87.89	4.32	**
SNA angle [°]	83.21	2.91	78.94	2.90	**
SNB angle [°]	78.56	2.79	74.24	2.46	**
ANB angle [°]	4.66	1.80	4.71	2.33	NS
ANB_ind_ [°]	3.68	1.34	3.49	1.48	NS
Calculated_ANB (ANB – ANB_ind_) [°]	0.97	1.64	1.20	1.65	NS
SN-Ba angle [°]	130.19	4.41	134.47	4.21	**
SN-Pg angle [°]	79.66	2.69	75.12	2.54	**
S–N (mm)	75.00	71.28	66.31	4.38	NS
Go-Me (mm)	74.94	67.54	65.24	5.33	*
Wits appraisal (mm)	2.00	3.96	0.71	4.89	**
ML-NSL angle [°]	27.79	4.69	35.38	4.90	**
+ 1/NL angle [°]	68.05	10.46	70.75	6.79	**
+ 1/SNL angle [°]	74.40	10.53	80.15	7.15	**
+ 1/NA angle [°]	22.38	10.60	20.90	7.09	NS
+ 1/NA (mm)	3.71	5.44	2.73	2.51	**
−1/ML (anatomic)	81.22	6.57	83.90	7.50	**
−1/NB angle [°]	25.12	6.99	25.72	7.30	NS
−1/NB (mm)	4.44	6.13	4.27	2.58	NS
Interincisal angle [°]	127.84	13.84	128.67	11.27	NS

We repeated the same clustering analysis with skeletal classes I and II, separately. Among skeletal class I patients, three clusters analysis was acceptable according to the dendrogram (Supplementary Fig. 4). The Ward’s method results showed that Cluster 1 comprised 105 patients, compared to 94 in Cluster 2 and 147 in Cluster 3. In addition, the three clusters of class I varied significantly in the cephalometric parameters, and among these parameters were the most critical parameters for diagnosing skeletal malocclusion ANB, Calculated_ANB, and Wits appraisal; detailed information is available in Table 3.

**Table 3:** Shows the results of the hierarchical clustering analysis for skeletal class I patients. Cephalometric parameters, and age, descriptive statistics (mean, and standard deviation (SD)) for each cluster. Besides, the table presents the significance levels between the three clusters using ANOVA test (NS—not significant, * < 0.05, and ** < 0.01). The three clusters of class I varied significantly in the cephalometric parameters, and among these parameters were the most critical parameters for diagnosing skeletal malocclusion ANB, Calculated_ANB, and Wits appraisal

Parameter	Class I -Occlusion						
	**Cluster 1**		**Cluster 2**		**Cluster 3**		
	Mean	SD	Mean	SD	Mean	SD	**Sig ANOVA**
Age	13.03	5.22	12.11	2.50	13.45	3.66	NS
NL-ML angle [°]	24.00	5.38	28.66	5.04	20.94	4.23	**
NL-NSL angle [°]	9.54	2.85	7.01	3.22	6.00	3.50	**
PFH/AFH (%)	64.59	3.96	63.48	3.97	70.49	4.00	**
Gonial angle [°]	123.12	5.62	126.05	5.38	120.33	5.14	**
Facial axis	89.13	3.93	88.10	4.13	92.65	3.59	**
SNA angle [°]	77.88	3.03	81.47	2.44	83.81	3.02	**
SNB angle [°]	75.11	2.32	76.46	2.11	80.32	2.15	**
ANB angle [°]	2.77	1.38	5.00	1.16	3.49	1.46	**
ANB_ind_ [°]	2.70	1.22	4.54	1.16	3.75	1.24	**
Calculated_ANB (ANB – ANB_ind_) [°]	0.07	0.85	0.41	0.78	−0.26	0.75	**
SN-Ba angle [°]	133.77	4.36	132.01	4.54	129.88	4.77	**
SN-Pg angle [°]	76.23	2.54	76.90	2.12	81.35	2.11	**
S–N (mm)	66.62	4.68	79.67	91.64	66.90	4.30	NS
Go-Me (mm)	66.01	5.52	78.63	89.69	68.36	5.30	NS
Wits appraisal (mm)	−0.55	2.26	0.30	5.26	0.33	2.08	*
ML-NSL angle [°]	33.56	4.66	35.67	5.09	26.93	4.29	**
+ 1/NL angle [°]	72.25	6.83	67.08	6.47	65.91	8.62	**
+ 1/SNL angle [°]	81.80	6.45	74.09	6.16	71.91	8.38	**
+ 1/NA angle [°]	20.32	7.49	24.44	6.52	24.27	8.61	**
+ 1/NA (mm)	2.90	2.56	4.90	6.68	4.05	2.56	NS
−1/ML (anatomic)	87.49	6.91	82.57	5.66	81.99	6.19	**
−1/NB angle [°]	21.18	6.77	29.56	4.70	25.27	6.49	**
−1/NB (mm)	2.66	2.17	6.75	6.72	3.64	2.10	NS
Interincisal angle [°]	135.74	12.19	120.99	8.74	126.97	10.82	**

Lastly, the skeletal class II dendrogram revealed that two cluster analyses were suitable for presenting the differences between the clusters within skeletal class II patients (Supplementary Fig. 5). The Ward’s method results showed that cluster 1 consisted of 88 patients, compared to 122 in cluster 2, as shown in Table 1 and Supplementary Fig. 5. In addition, the two clusters interestingly varied significantly in many cephalometric parameters- NL-ML angle, PFH/AFH ratio, Gonion angle, Go-Me (mm), ML-NSL, −1/NB angle, −1/NB (mm), and interincisal angle, as presented in Table 4.

**Table 4:** Shows the results of the hierarchical clustering analysis for skeletal class II patients—cephalometric parameters, and age, descriptive statistics (mean and standard deviation (SD)) for each cluster. Besides, the table presents the significance levels between the two clusters using t-test analysis significance levels of comparisons between the two clusters for each parameter (NS—not significant, * < 0.05, and ** < 0.01). The two clusters interestingly variated significantly in many cephalometric parameters- NL-ML angle, PFH/AFH, gonial angle, Go-Me (mm), ML-NSL, −1/NB angle, −1/NB (mm), and interincisal angle

Parameter	Class II Malocclusion				
	**Cluster 1**		**Cluster 2**		
	Mean	SD	Mean	SD	**Sig t test**
Age	12.67	4.70	13.44	7.73	NS
NL-ML angle [°]	18.80	4.73	25.72	5.21	**
NL-NSL angle [°]	7.19	3.24	9.33	3.31	**
PFH/AFH (%)	71.31	3.61	63.74	4.01	**
Gonial angle [°]	114.85	5.13	122.70	6.12	**
Facial axis	90.92	3.49	86.96	4.23	**
SNA angle [°]	81.98	3.44	80.32	2.90	**
SNB angle [°]	76.23	2.56	73.59	2.63	**
ANB angle [°]	5.75	1.49	6.73	1.63	**
ANB_ind_ [°]	2.83	1.36	3.98	1.26	**
Calculated_ANB (ANB – ANB_ind_) [°]	2.92	1.12	2.75	1.05	NS
SN-Ba angle [°]	131.24	4.46	134.35	4.29	**
SN-Pg angle [°]	77.92	2.32	74.39	2.70	**
S–N (mm)	66.73	5.96	76.04	77.36	NS
Go-Me (mm)	66.45	6.51	73.40	70.94	NS
Wits appraisal (mm)	4.10	2.68	3.28	6.37	NS
ML-NSL angle [°]	25.99	3.89	35.06	4.67	**
+ 1/NL angle [°]	71.63	13.35	70.86	7.31	NS
+ 1/SNL angle [°]	78.83	13.79	80.19	7.88	NS
+ 1/NA angle [°]	19.19	13.76	19.48	7.35	NS
+ 1/NA (mm)	1.87	4.03	2.33	4.75	NS
−1/ML (anatomic)	79.56	7.19	80.70	7.29	NS
−1/NB angle [°]	22.66	7.40	27.96	6.77	**
−1/NB (mm)	3.08	2.11	5.79	6.96	**
Interincisal angle [°]	132.41	17.35	125.83	9.32	**

### Machine learning models

**Fig. 1:**
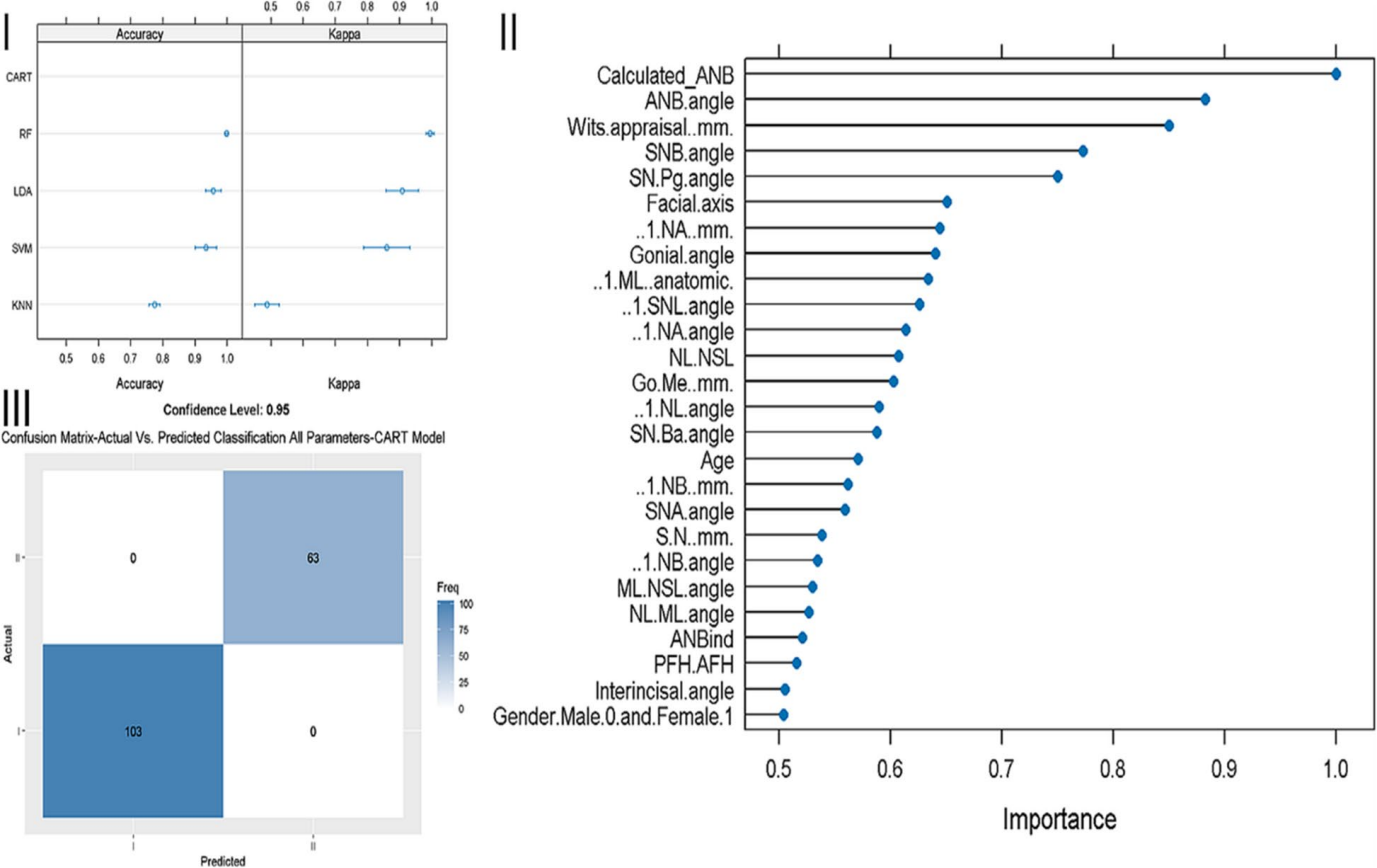
Evaluation of a general machine learning model, which included all cephalometric and demographic variables (gender, age). 1-I: Accuracy and reliability (kappa) of different machine learning models (RF, KNN, SVM, LDA, CART, GLM), The X-axis shows the Accuracy and Kappa scores (95% confidence interval), for each model. 1-II: Importance of each parameter in the machine learning model (RF), X-axis shows the prediction importance score of the assessed parameters. Y-axis shows the list of the assessed parameters. 1-III: Confusion matrix to demonstrate the sensitivity and specificity of the RF model in classifying patients as skeletal class I or II. The X-axis shows the class prediction, and the Y-axis shows the number of identified patients in each classification

Several machine learning models were evaluated regarding accuracy, reliability (kappa), sensitivity, and specificity in correctly classifying a patient as skeletal class I or II based on several input variables, including cephalometric and demographic (age, gender). First, all input variables (general model) were used to determine the performance of the models LDA, CART, KNN, SVM, RF, and GLM, which reached a mean accuracy of 95.64%, 100.0%, 88.24%, 93.38%, 99.74%, and 95.64%, respectively (Fig. 1-I). Then, the importance of each input variable on the machine learning model was evaluated using the RF model (Fig. 1-II). As evident from Fig. 1-II, the most critical variable was Calculated_ANB, followed by ANB and Wits appraisal. Finally, the sensitivity and specificity of the best models (CART, and RF) were tested, and according to Fig. 1-III, the model led to perfect sensitivity and specificity. The RF model was chosen for the calculations shown in Fig. 1-II and III due to its high accuracy (100%) compared to the other machine learning models.

Based on the findings in Fig. 1-II, further machine-learning models were established and evaluated concerning their accuracy, reliability (kappa), sensitivity, and specificity. In detail, the number of input variables was reduced by selecting only the most essential parameters, according to Fig. 1-II. Hence, models 1, 2, and 3 consisted of one, two, and three input variables, respectively, determined by the stepwise forward method (excluding the Calculated_ANB), starting at ANB. Thereby, three more models were generated and evaluated, which is summarized in Table 5.

**Table 5:** Machine-learning models results

Model	Best model	Hyperparameters	Cross validation accuracy	Cross validation kappa	Validation sensitivity	Validation specificity
General model: all cephalometric and demographic parameters	CART, RF		CART = 100%, RF = 99.74%	CART = 100%, RF = 99.45%	100%	100%
Model 1: ANB	KNN	K = 9	82.55%	62.71%	86.41%	69.84%
Model 2: ANB + Wits	KNN	K = 9	86.6%	71.42%	88.35%	76.19%
Model 3: ANB + Wits + SNB	KNN	K = 7	90.53%	79.53%	87.38%	79.37%
Model 4: SNA + SNB + ML-NSL	GLM		99.48%	98.89%	100%	98.41%

Performance of five machine learning models (general, 1, 2, 3, 4) in diagnosing skeletal I or II. The best-fitting model in terms of accuracy and kappa is reported for each model

According to the results presented in Table 5, the highest accuracy and reliability (kappa) was achieved with the general model (100%), but considering ANB only (model 1) resulted in an accuracy of 82.6% and a kappa of 62.7%. Adding the Wits appraisal (model 2) led to better performance of the machine learning model, although remarkably better values than model 1 were noticed in model 3. Here, adding the Wits appraisal and SNB resulted in an accuracy of 90.53% and a kappa of 79.53% in the KNN model.

**Fig. 2:**
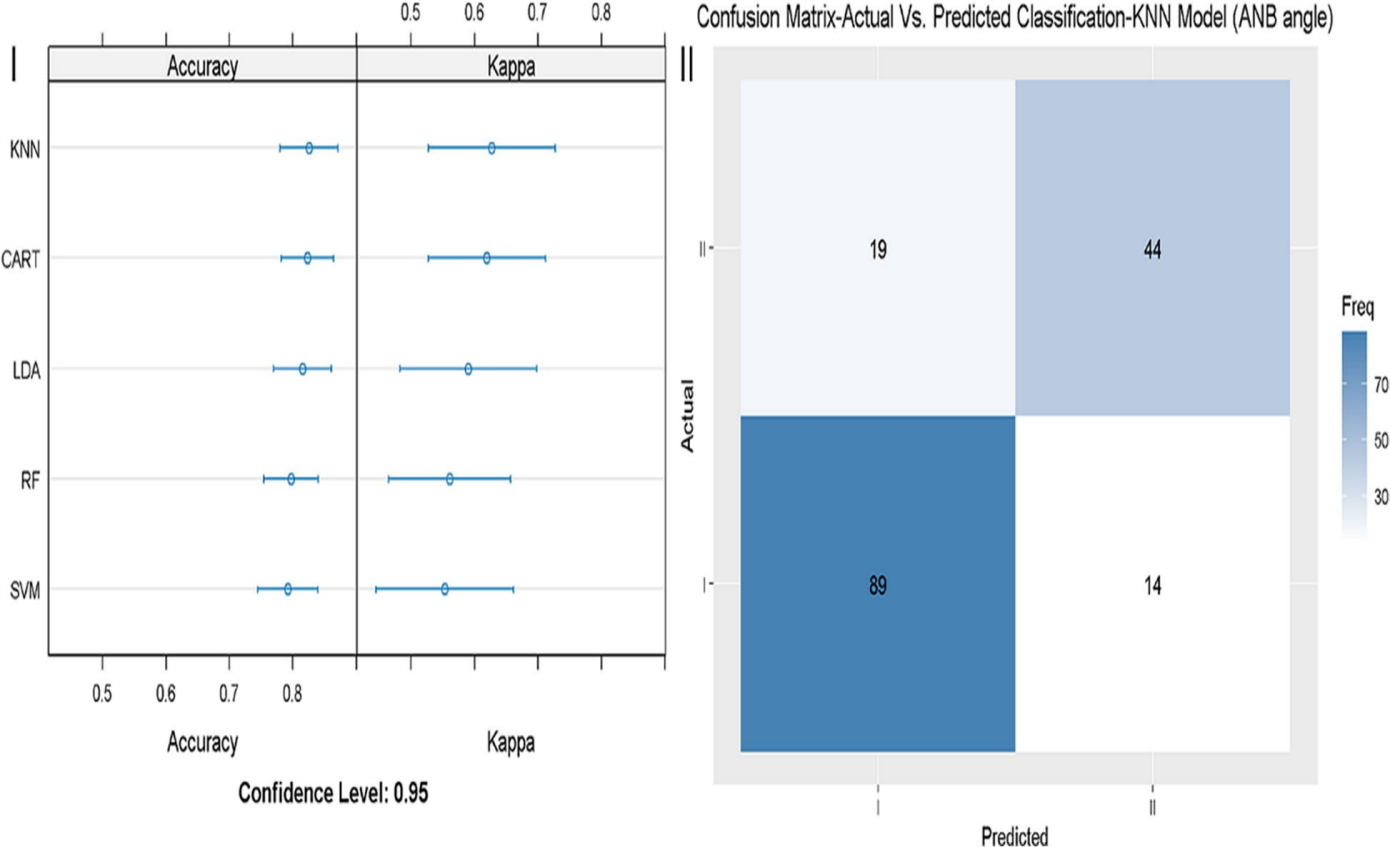
Evaluation of the machine learning model 1 (ANB only). 2-I: different models were tested (RF, KNN, SVM, LDA, CART, GLM), The X-axis shows the Accuracy and Kappa scores (95% confidence interval), for each model. 2-II: sensitivity and specificity of the best fitting model 1 (KNN) in diagnosing skeletal class I and II. The X-axis shows the class prediction, and the Y-axis shows the number of identified patients in each classification

In Fig. 2**,** the performance of model 1, which included ANB only, is demonstrated. Among the different models tested, KNN showed the highest accuracy and kappa (Fig. 2-I). In contrast to the perfect sensitivity and specificity (100%) achieved in the general model, those parameters were lower in model 1, i.e., 86.4% and 69.8%, respectively, illustrated in Fig. 2-II.

**Fig. 3:**
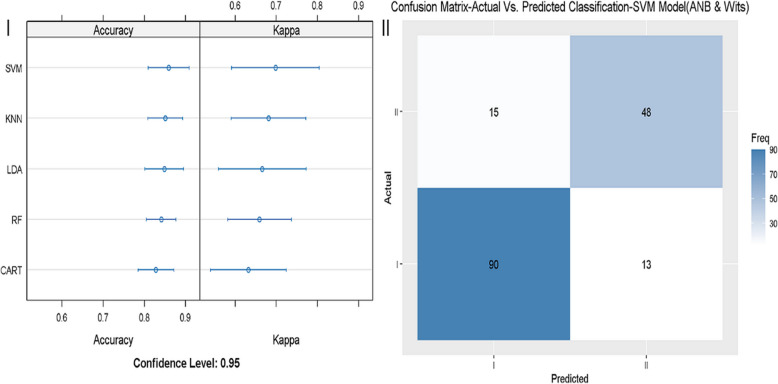
Evaluation of the machine learning model 2 (ANB + Wits). 3-I: different models were tested (RF, KNN, SVM, LDA, CART, GLM), The X-axis shows the Accuracy and Kappa scores (95% confidence interval), for each model. 3-II: sensitivity and specificity of the best fitting model 2 (SVM) in diagnosing skeletal class I and II. The X-axis shows the class prediction, and the Y-axis shows the number of identified patients in each classification

**Fig. 4:**
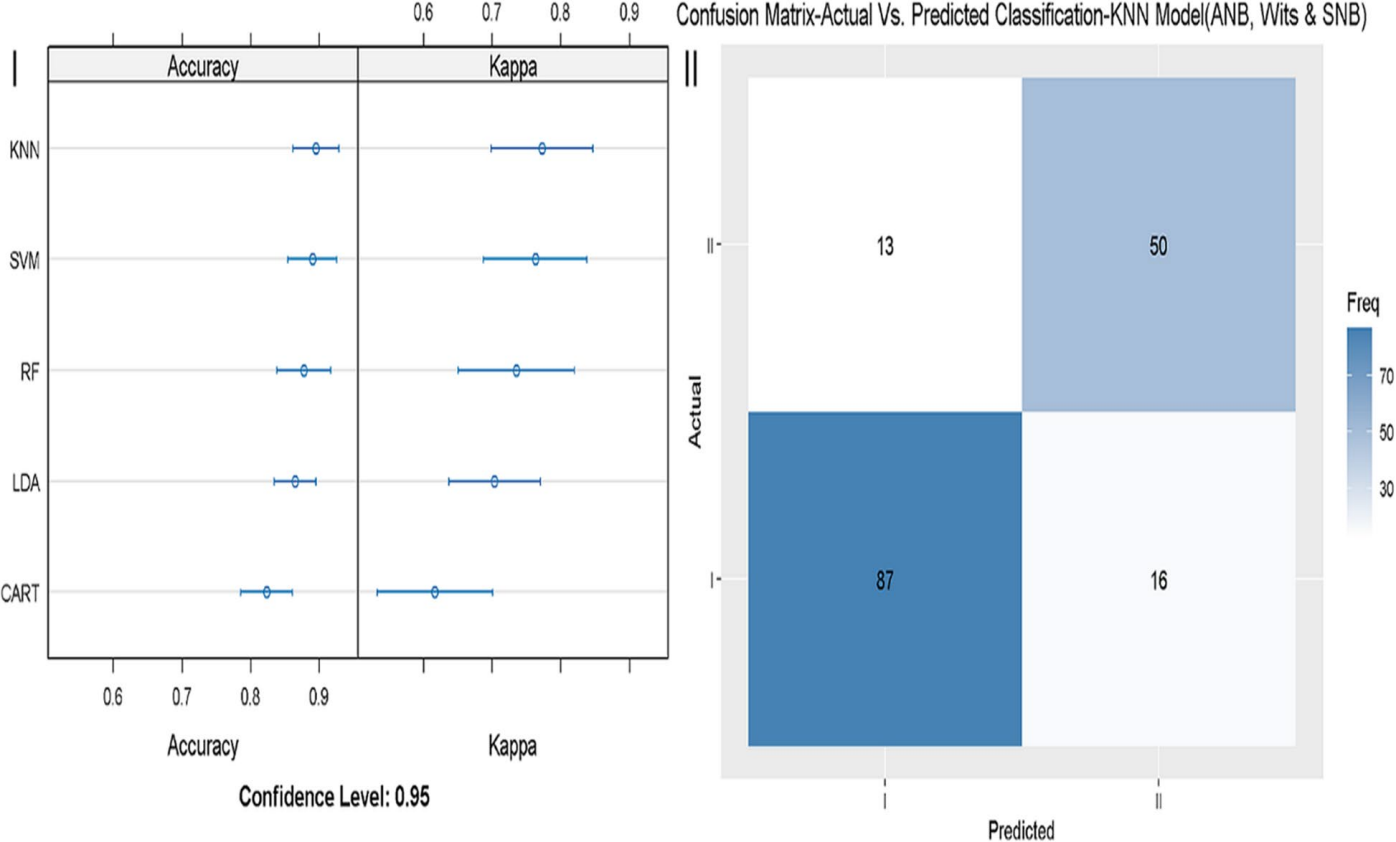
Evaluation of the machine learning model 3 (ANB + Wits + SNB). 4-I: different models were tested (RF, KNN, SVM, LDA, CART, GLM), The X-axis shows the Accuracy and Kappa scores (95% confidence interval), for each model. 4-II: sensitivity and specificity of the best fitting model 3 (KNN) in diagnosing skeletal class I and II. The X-axis shows the class prediction, and the Y-axis shows the number of identified patients in each classification

**Fig. 5:**
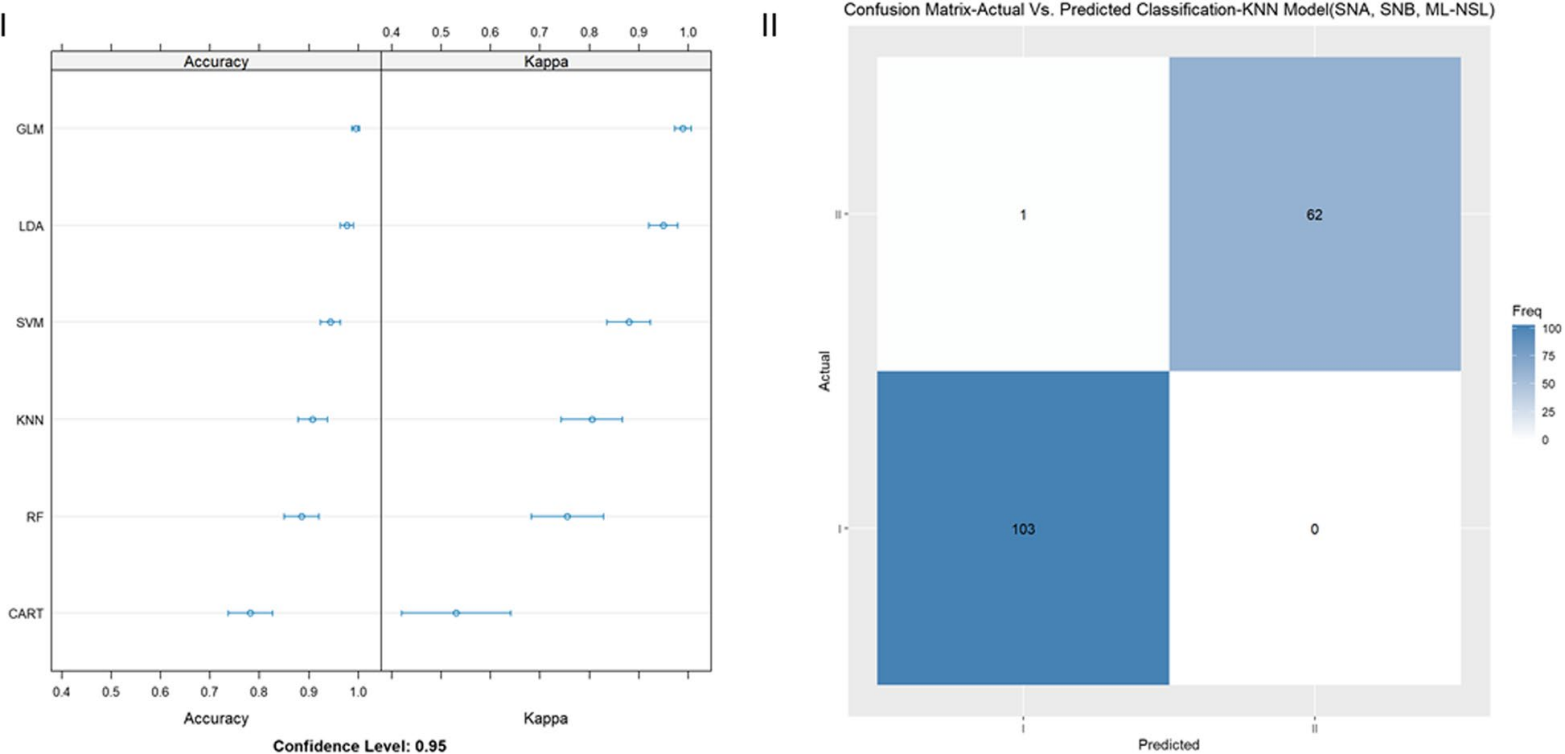
Evaluation of the machine learning model 4 (SNA + SNB + ML-NSL). 5-I: different models were tested (RF, KNN, SVM, LDA, CART, GLM), The X-axis shows the Accuracy and Kappa scores (95% confidence interval), for each model. 5-II: sensitivity and specificity of the best fitting model 3 (KNN) in diagnosing skeletal class I and II. The X-axis shows the class prediction, and the Y-axis shows the number of identified patients in each classification

The accuracy, kappa, sensitivity, and specificity of model 2, in which ANB and Wits appraisal were considered, are presented in Fig. 3. The best accuracy and reliability were reached by the model KNN (Fig. 3-I), and sensitivity (88.3%) and specificity (76.2%) were slightly higher than in model 1 (Fig. 3-II). In model number 3, which incorporated the parameters ANB, Wits appraisal, and SNB, the best-fitting model was KNN (Fig. 4-I). Sensitivity and specificity reached 87.38% and 79.37%, respectively, which could be a more evident improvement than model 2 (Fig. 4-II). Finally, we applied a machine-learning model (model 4) that included the parameters that define the ANB angle and the Calculated_ANB defined by Panagiotidis and Witt [13] (i.e., SNA, SNB, and ML-NSL angles). This model demonstrated a significant improvement in accuracy, up to 99.48% in the GLM model (Table 5). The GLM model showed perfect sensitivity in the validation data (100%), and almost perfect specificity (98.41%) (Fig. 5I-II).

## Discussion

Our study aimed to reveal new information about German orthodontic patients using hierarchical clustering and machine learning methods to correctly classify individual orthodontic patients as skeletal class I or II, testing different models in terms of the type of machine learning (RF, KNN, SVM, LDA, CART) and input data (general model, models 1 to 3).

The first step in this study was to perform hierarchical clustering that revealed distinct characteristics for each cluster within all data and for each patient group (skeletal class I/II) separately, based on various cephalometric parameters. We found that among skeletal class I patients, it’s acceptable to apply three clusters analysis. The Ward’s method results showed that Cluster 1 was comprised of 105 patients, compared to 94 in Cluster 2 and 147 in Cluster 3. In addition, the three clusters of class I variated significantly in the cephalometric parameters, and among these parameters were the most critical parameters for the diagnosis of skeletal malocclusion ANB, Calculated_ANB, and Wits appraisal.

Skeletal class II dendrogram revealed that the two clusters analysis was good for presenting the differences between the clusters within skeletal class II patients. The Ward’s method results showed that cluster 1 consisted of 88 patients, compared to 122 in cluster 2. In addition, the two clusters interestingly variated significantly in many cephalometric parameters- NL-ML angle, PFH/AFH ratio, Gonion angle, Go-Me (mm), ML-NSL, −1/NB angle, −1/NB (mm), and interincisal angle. A study of Uribe et al. about phenotypic diversity in white adults with Class II malocclusion found that models with 2, 3, or 4 clusters were statistically acceptable. Still, they identified five distinct Class II phenotypes [30]. Another study that applied Cluster analysis to Class I occlusion found that the grouping pattern in Class I occlusion is shown in younger age levels and disappears with age. Also, they found that the clustering pattern is very similar in males and females with Class I [31].

The machine learning models analyzed varied in terms of the input variables (general model, models 1 to 3) and the technical method (LDA, CART, KNN, SVM, RF, and GLM), resulting in different performances, which was measured by mean accuracy, kappa, sensitivity, and specificity. In the general model, the highest accuracy (100%) and kappa (100%) were achieved by the CART model, and almost by the RF model (Accuracy = 99.74%, Kappa = 99.45%), whereas KNN was the best fitting model for models 1 (ANB only), model 2 (ANB, and Wits), and model 3 (ANB, Wits and SNB). Finally, in model 4 (SNA, SNB, and Wits), the GLM model demonstrated almost perfect accuracy (99.48%), and Kappa (98.89%). Thus, depending on the input variables and the desired outcome, different models should be applied to achieve the best performance. For example, in another study that was done by Zhou et al. 2023 [32], which was conducted to automatically determine an individual’s skeletal class and vertical facial growth using image processing, feature engineering, grid search, and cross validation, and nine different machine learning models were tested (KNN, Gaussian NB, the multi-layer perceptron (MLP), linear SVM, Gaussian process classifier, extreme gradient boosting, adaptive boosting, quadratic discriminant analysis, RF): whereas the model MLP was the best fitting model for the diagnosis of skeletal class (97.56% accuracy), the model linear SVM achieved the highest accuracy (90.24%) in determining an individual’s vertical facial pattern [32]. Further investigations revealed not only perfect accuracy and reliability, but also 100% sensitivity and specificity for the general CART, and RF models. Besides the perfect performance of the general model, attempts were made to simplify the model by reducing the number of input variables choosing the most important ones. Hence, in the general model, further analyses revealed Calculated_ANB, ANB, Wits appraisal and SNB to be the most important variables for the machine learning model. However, according to this analysis sagittal parameters, including the mandible’s degree of prognathism (SNB), appear to dominate vertical ones in the diagnosis of skeletal class I and II.

Comparing the models 1, 2, 3 and 4, the increase in performance between model 1 and 2 (+ 4.05% accuracy) can be regarded as rather irrelevant, whereas the rise observed between models 1 and 4 (+ 16.93% accuracy) appears to be more clinically relevant. Comparing validation of the different models, sensitivity and specificity varied between models 1 to 4: the highest sensitivity, and specificity were received in model 4 (Sensitivity = 100%, Specificity = 98.41%). Thus, evaluating all five models, model 4, which considers SNA, SNB and ML-NSL, could be applied in daily routine, because of the noticeable reduction in input information and still high and precise performance. Our results in this study overcome the results that were recently published by Midlej et al. [25], that included orthodontic Arab skeletal class I, and II patients, found that machine-learning model that included all parameters for patient classification showed a classification accuracy of 0.87 in the RF model, and the Classification and Regression Tree models. The same study also found that using ANB angle and Wits appraisal only gained an accuracy of 0.78 [25].

This study considered only German orthodontic patients to account for differences in cephalometry due to ethnicity. During patients’ recruitment, ancestry was not asked for, which might have resulted in a study collective that consists of other ethnic groups too. However, due to the German location of all study centers a German population can be assumed. Another limitation might be the heterogeneity in numbers of the two groups and the subgroups within each class. This factor can be explained by the methods applied, since patients were retrospectively allocated into specific (sub) groups. Future investigations, however, should aim to match the numbers across different (sub) groups. Also, potential errors in the identification of reference landmarks during cephalometric analysis might be considered as a limiting factor. But, according to statistical tests, high interrater and intrarater reliability have been proven in advance, allowing for reproducible measurements. Although, this is not the first study in this field, however, it’s to our knowledge it’s the first to be done on German population, and because of the variance between ethnic groups, it’s crucial to validate these models on this population. Furthermore, this study demonstrates a straightforward simple and accurate process, which is not always the case in other studies that used for example image processing, and feature engineering, which might be complicated to apply in the standard of care. Finally, due to concerns that are still available among orthodontists regarding the usage of machine-learning models in the diagnosis process, this study demonstrated the power of this tool.

## Conclusion

This research revealed new information regarding the distinct characteristics for each cluster within all data and for each patient group (skeletal class I/II) separately. Although age and gender are confounding factors influencing cephalometric measurements, they appear not to be important variables for skeletal class diagnosis in machine learning models. The GLM method, applied in a model considering SNA, SNB, and Wits appraisal only demonstrated 99.48% accuracy, and could be more accurate than the traditional equation used nowadays. In addition, by incorporating the machine-learning models suggested in this study, orthodontic practitioners can save valuable time and effort in the diagnosis process by focusing only on specific parameters and without the need for matching any equations that can misclassify borderline cases. We believe that the use of the models suggested in this study can contribute to precise, personalized diagnosis and treatment planning. Furthermore, the relevance of those cephalometric parameters in the machine learning model illustrates the importance of accurate and reliable identification of the corresponding landmarks. Future investigations should aim to match sample sizes of the age subgroups and validate these findings in larger, new populations.

## Supplementary Information

Supplementary Material 1: Supplementary Table 1. List and definition of the relevant skeletal and dental cephalometric variables analyzed. Supplementary Table 2 A. Demographic characteristics of the study groups and distribution to the subgroups. *n* = absolute numbers, % = relative frequency. Supplementary Table 2B. Cephalometric measurements of patients with skeletal class I and II according to the definition of Panagiotidis and Witt. M = Mean, SD = Standard deviation, Min = Minimum, Pctl. 25 = 25 % percentile, Pctl. 75 = 75 % percentile, Max = Maximum. Supplementary Fig. 1 A-B. Reference lines and marks needed for cephalometric evaluation. Angle 1 = Facial axis. Details are described in Table 1. Supplementary Fig. 2. Range of Calculated_ANB-values with the corresponding density in different age and gender-specific subgroups. Supplementary Fig. 3. This figure represents the hierarchical clustering results for skeletal class I and II patients. The two colors represent the two distinct clusters. Patients’ cephalometric parameters that are close to each other are connected with vertical lines. Supplementary Fig. 4. This figure represents the hierarchical clustering results for skeletal class I patients. The three colors represent the distinct three clusters. Patients’ cephalometric parameters that are close to each other are connected with vertical lines. Supplementary Fig. 5. This figure represents the hierarchical clustering results for skeletal class II patients. The three colors represent the distinct three clusters. Patients’ cephalometric parameters that are close to each other are connected with vertical lines.

## Data Availability

The datasets used and analyzed during the current study are available from the corresponding author on reasonable request.
