# Supplementary material for: Hierarchical clustering analysis & machine learning models for diagnosing skeletal classes I and II in German patients
Source: BMC Oral Health. 2025 May 15;25:731. doi: 10.1186/s12903-025-06063-6 (PMC12083114; doi:10.1186/s12903-025-06063-6)

Supplementary Table 1. List and definition of the relevant skeletal and dental cephalometric variables analyzed.

variable	definition
**skeletal sagittal**	
SNA [°]	caudal angle between Sella (S), Nasion (N) and point A (A)
SNB [°]	caudal angle between Sella, Nasion and point B (B)
ANB [°]	caudal angle between Nasion, point A and point B)
Wits appraisal [mm]	anterior-posterior distance between perpendiculars through A (A') and B (B') at occlusal plane (Occl)<br>< 0:B' is anterior to A'<br>≥ 0: B' is posterior to A'
SN-Ba [°]	caudal angle between Sella, Nasion and Basion (Ba)
SN-Pg [°]	caudal angle between Sella, Nasion and Pogonion (Pg)
S-N [mm]	distance between Sella and Nasion; length of the anterior cranial base
Go-Me [mm]	distance between Gonion (Go) and Menton (Me); length of mandibular plane (ML)
**skeletal vertical/ growth**	
NL/NSL	anterior angle between Sella-Nasion-line (NSL) and nasal line (NL)
ML/NSL	anterior angle between NSL an ML
NL/ML	anterior angle between the NL and ML
PFH/AFH	ratio between posterior (S-Go) and anterior (N-Me) facial height; growth pattern
Gonion angle	anterior angle between ML and line Go-Ar; growth pattern
Facial axis	posterior-caudal angle between the lines N-Ba and Pt-GN; growth pattern
**Dental**	
+1/NL [°]	anterior-caudal angle between upper incisors' tooth axis (+1) and NL
+1/NSL [°]	anterior-caudal angle between +1 and NSL
+1/NA [°]	caudal angle between upper incisors' tooth axis and line NA
+1i/NA [mm]	perpendicular distance between line NA and upper incisal point (+1i)
-1/ML [°]	anterior-cranial angle between lower incisors' tooth axis and line ML
-1/NB [°]	caudal angle between lower incisors' tooth axis and line NB
-1i/NB [mm]	perpendicular distance between NB and lower incisal point (-1i)
Interincisal_angle [°]	posterior angle between +1 and -1

Supplementary Table 2A. Demographic characteristics of the study groups and distribution to the subgroups. n = absolute numbers, % = relative frequency.

group	(n, %)	age (years)	age: ≤ 13 (n, %)	age: 14-21 (n, %)	age: > 21 (n, %)	Gender	
						female (n, %)	male (n, %)
class I	346, 62	13 ± 4	275, 79	57, 16	14, 4	194, 56	152, 44
class I female	-	-	154, 45	32, 9	8, 2	-	-
class I male	-	-	121, 35	25, 7	6, 2	-	-
class II	210, 38	13 ± 6.6	164, 78	33, 16	13, 6	125, 60	85, 40
class II female	-	-	96, 46	22, 10	7, 3	-	-
class II male	-	-	68, 32	11, 5	6, 3	-	-

Supplementary Table 2B. Cephalometric measurements of patients with skeletal class I and II according to the definition of Panagiotidis and Witt. M = Mean, SD = Standard deviation, Min = Minimum, Pctl. 25 = 25 % percentile, Pctl. 75 = 75 % percentile, Max = Maximum.

**variable**	**M**		**SD**		**Min**		**Pctl. 25**		**Pctl. 75**		**Max**	
**skeletal class**	**I**	**II**	**I**	**II**	**I**	**II**	**I**	**II**	**I**	**II**	**I**	**II**
SNA [°]	81	81	3.8	3.2	63	73	79	79	84	83	92	89
SNB [°]	78	75	3.2	2.9	66	66	76	73	80	77	87	82
SN-Pg [°]	79	76	3.3	3.1	67	67	76	74	81	78	88	85
ANB [°]	3.7	6.3	1.6	1.6	-2.5	1.7	2.7	5.2	4.7	7.4	9	12
Wits appraisal [mm]	0.057	3.6	3.3	5.2	-6	-5	-1.3	1.9	1.7	5.3	8.6	13
ANB individual [°]	3.6	3.5	1.4	1.4	-3.2	-0.5	2.8	2.6	4.6	4.5	7.8	8.1
Calculated ANB [°]	0.025	2.8	0.84	1.1	-1.5	1.5	-0.68	2	0.78	3.4	1.5	8.6
S-N [mm]	70	72	48	59	42	57	64	63	69	69	81	107
Go-Me [mm]	70	70	47	54	43	54	63	61	70	69	85	100
SN-Ba [°]	132	133	4.9	4.6	117	123	129	130	135	137	144	145
NL/ML [°]	24	23	5.7	6.1	5.3	4	20	19	27	27	46	42
NL/NSL [°]	7.4	8.4	3.6	3.4	-3.1	-1.8	5.1	6.1	9.8	10	18	19
ML/NSL [°]	31	31	6	6.3	16	13	27	27	35	35	52	47
PFH.AFH [%]	67	67	5.1	5.4	50	54	63	63	71	71	80	85
Gonion_Angle [°]	123	119	5.8	6.9	101	104	119	114	126	124	139	139
Facial_Axis [°]	90	89	4.3	4.4	76	71	88	86	93	91	101	99
+1/NL [°]	68	71	8	10	37	46	64	64	73	78	94	113
+1/NSL [°]	76	80	8.4	11	43	53	71	73	81	87	98	122
+1/NA [°]	23	19	7.9	10	2.2	-22	18	12	28	26	54	47
+1i/NA [mm]	3.9	2.1	4.2	4.5	-2.4	-12	1.9	0.025	5.3	4.3	11	10
-1/ML [°]	84	80	6.7	7.3	65	58	79	76	88	84	105	104
-1/NB [°]	25	26	6.9	7.5	-1.1	4	21	21	30	31	44	41
-1i/NB [mm]	4.2	4.7	4.3	5.6	-3.3	-2.5	2.2	2.6	5.6	5.8	12	12
Interincisal_Angle [°]	128	129	12	14	96	89	120	119	135	135	172	187

Supplementary Figure 1. Reference lines and marks are needed for cephalometric evaluation. Angle 1 = Facial axis. Details are described in Table 1.

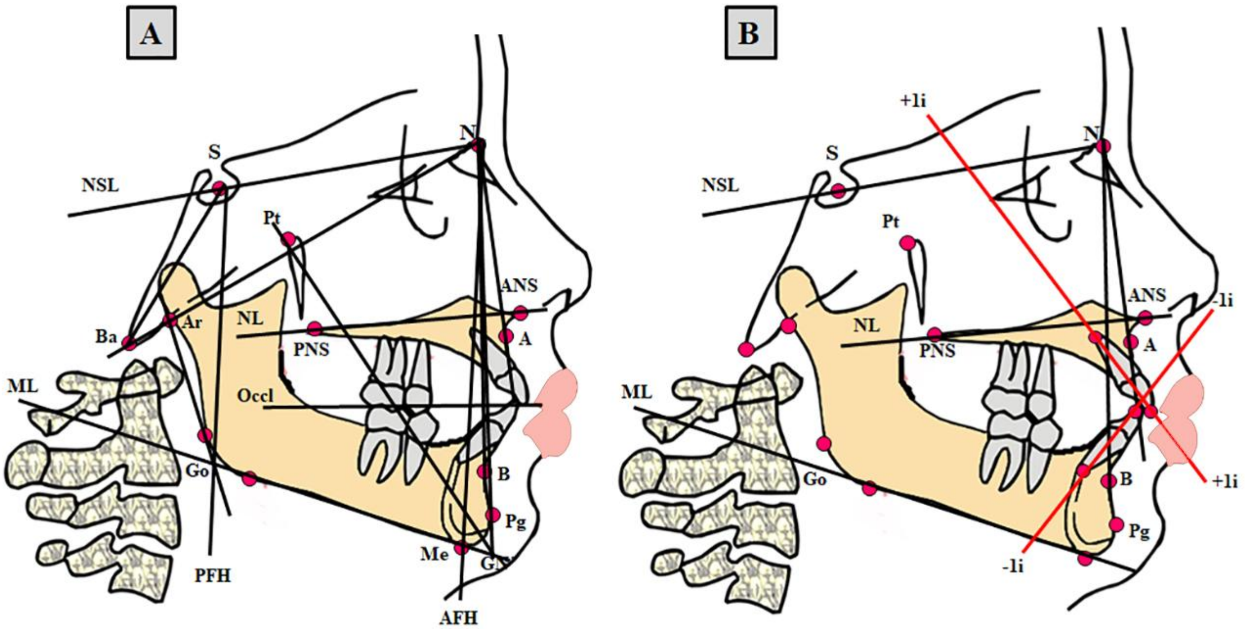

Supplementary Figure 2.

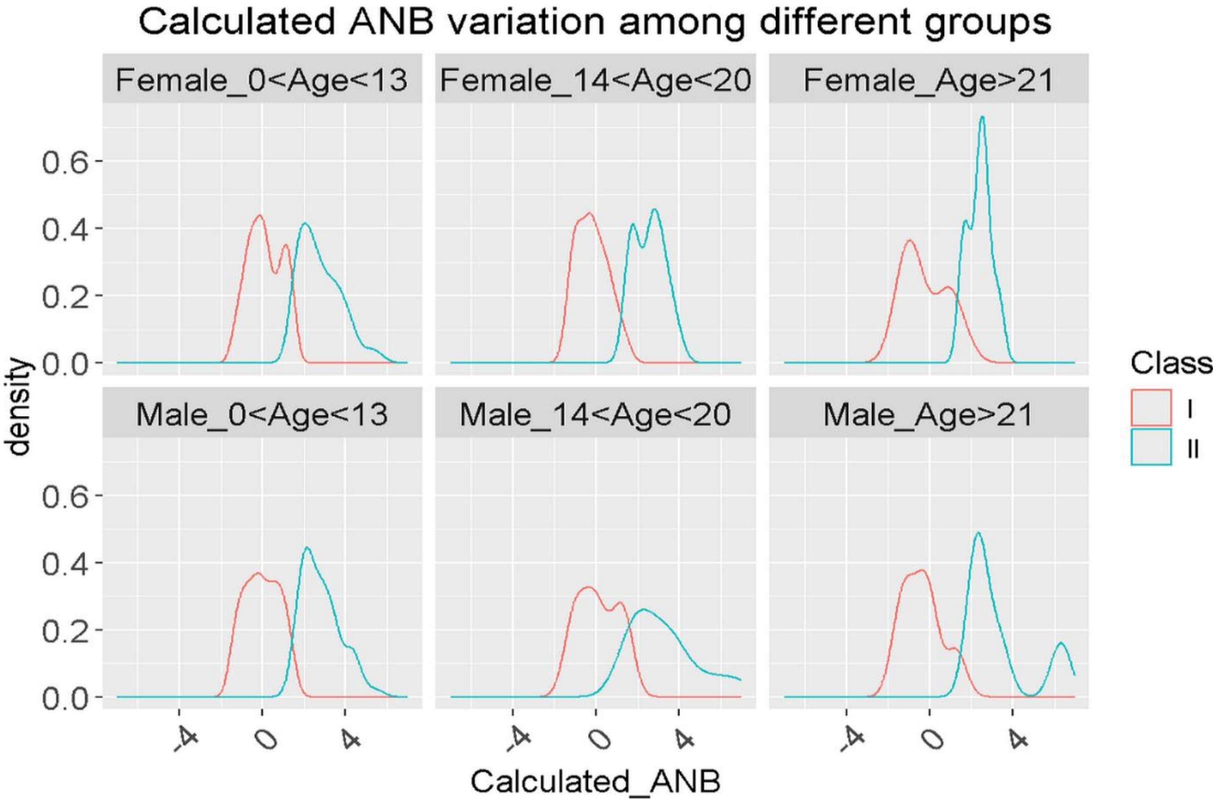

Supplementary Figure 3.

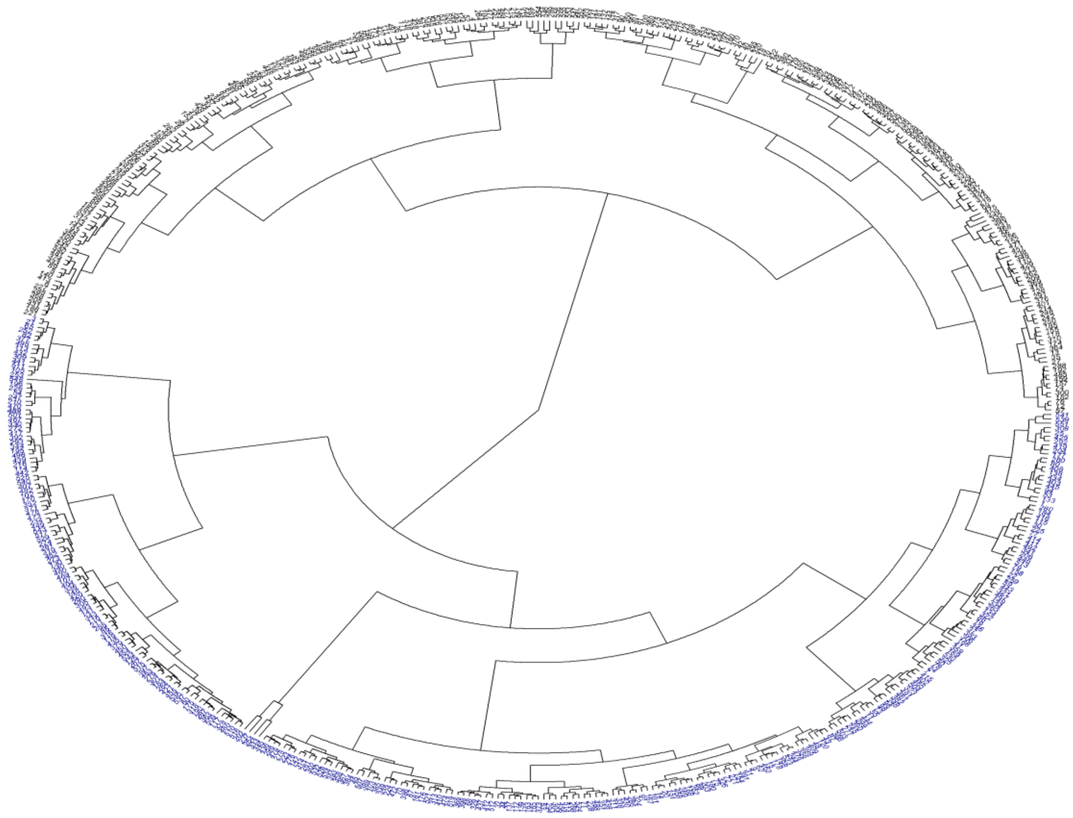

Supplementary Figure 4.

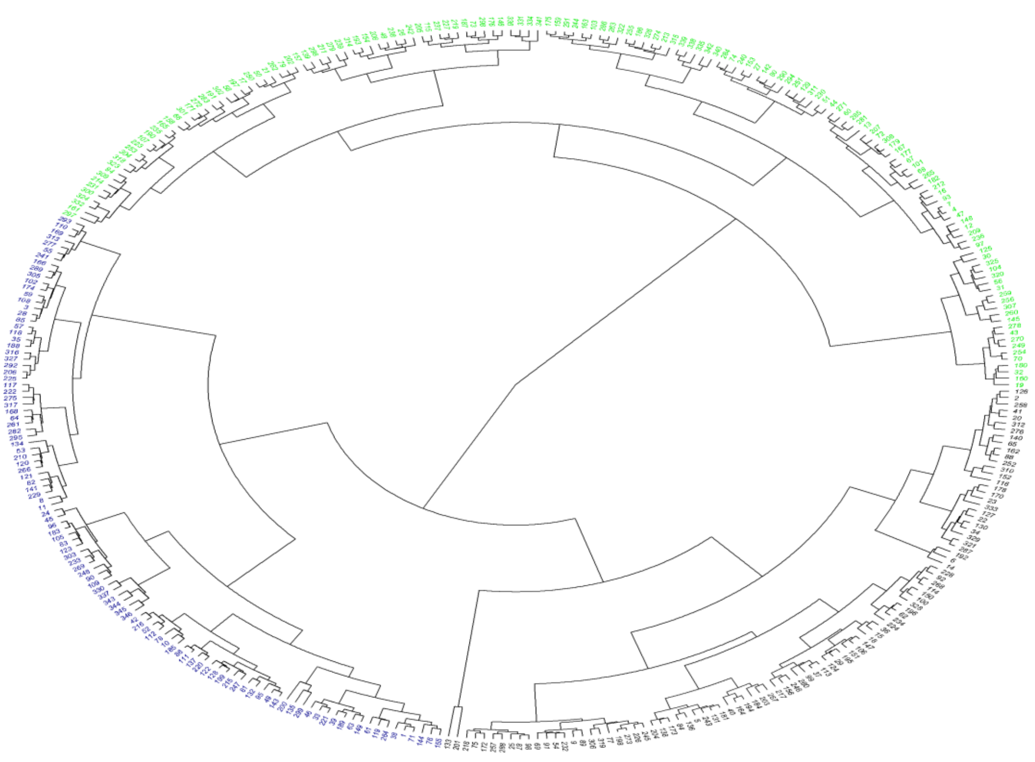

Supplementary Figure 5.

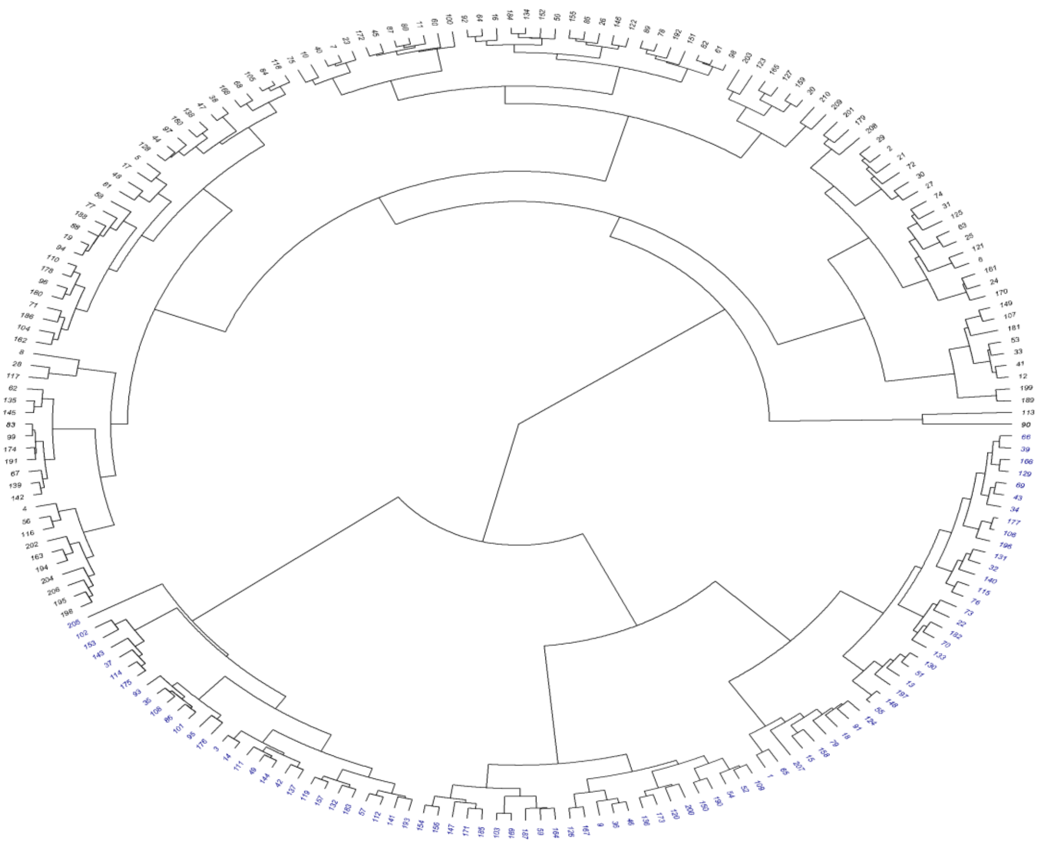

Supplement: Supplementary file 1 — Supplementary Material 1: Supplementary Table 1. List and definition of the relevant skeletal and dental cephalometric variables analyzed. Supplementary Table 2 A. Demographic characteristics of the study groups and distribution to the subgroups. n = absolute numbers, % = relative frequency. Supplementary Table 2B. Cephalometric measurements of patients with skeletal class I and II according to the definition of Panagiotidis and Witt. M = Mean, SD = Standard deviation, Min = Minimum, Pctl. 25 = 25 % percentile, Pctl. 75 = 75 % percentile, Max = Maximum. Supplementary Fig. 1 A-B. Reference lines and marks needed for cephalometric evaluation. Angle 1 = Facial axis. Details are described in Table 1. Supplementary Fig. 2. Range of Calculated_ANB-values with the corresponding density in different age and gender-specific subgroups. Supplementary Fig. 3. This figure represents the hierarchical clustering results for skeletal class I and II patients. The two colors represent the two distinct clusters. Patients’ cephalometric parameters that are close to each other are connected with vertical lines. Supplementary Fig. 4. This figure represents the hierarchical clustering results for skeletal class I patients. The three colors represent the distinct three clusters. Patients’ cephalometric parameters that are close to each other are connected with vertical lines. Supplementary Fig. 5. This figure represents the hierarchical clustering results for skeletal class II patients. The three colors represent the distinct three clusters. Patients’ cephalometric parameters that are close to each other are connected with vertical lines. [file 12903_2025_6063_MOESM1_ESM.pdf]
